# A SNaPshot assay for the rapid and simple detection of four common hotspot codon mutations in the *PIK3CA *gene

**DOI:** 10.1186/1756-0500-2-66

**Published:** 2009-04-29

**Authors:** Carolyn D Hurst, Tahlita CM Zuiverloon, Christian Hafner, Ellen C Zwarthoff, Margaret A Knowles

**Affiliations:** 1 Cancer Research UK Clinical Centre, Leeds Institute of Molecular Medicine, St James's University Hospital, Leeds, UK; 2 Department of Pathology, Josephine Nefkens Institute, Erasmus MC, Rotterdam, the Netherlands; 3 Department of Dermatology, University of Regensburg, 93042 Regensburg, Germany

## Abstract

**Background:**

Activating mutations in the *PIK3CA *gene have been identified in a variety of human malignancies and are commonly detected in hotspot codons located in the helical and kinase domains in exons 9 and 20. Existing methodologies for the detection of *PIK3CA *mutations are time-consuming and/or expensive. In the present study we describe the first application of a *PIK3CA *SNaPshot assay to the screening of frequent mutations in these exons.

**Findings:**

A SNaPshot assay for the simultaneous detection of four frequent PIK3CA hotspot mutations (E542K, E545G, E545K and H1047R) has been developed and evaluated. The assay combines multiplex PCR amplification with a multiplex primer extension assay to allow targeted detection of all four mutations in one reaction. The method was tested using samples that had previously been analysed for mutations by high-resolution melting analysis and sequencing. All mutations detected were concordant and no false positive results were obtained. Sensitivity tests showed that the SNaPshot assay could detect mutant DNA when it represents 5–10% of the total DNA present. The application of the method to the analysis of DNAs extracted from formalin-fixed paraffin-embedded samples was also demonstrated.

**Conclusion:**

The SNaPshot assay described here offers a fast, sensitive, inexpensive and specific approach to the analysis of frequent *PIK3CA *mutations in both fresh and archival patient samples.

## Background

The phosphatidylinositol 3-kinase (PI3K) pathway plays an important role in many cellular processes including cell proliferation, adhesion, survival and motility. Dysregulation of this pathway has been observed in many types of human malignancy and has commonly been associated with genetic alterations in components of the pathway (reviewed in [1]). Such genetic alterations include activating mutations in the *PIK3CA *gene encoding the p110α subunit of class IA PI3K.

Somatic mutations of *PIK3CA *have now been reported in several types of human cancer [2-13]. Although mutations have been detected throughout the *PIK3CA *gene, common mutational hotspots occur in the helical (exon 9) and kinase (exon 20) domains with E542K, E545K and H1047R being most frequent. Recently, mutations of *PIK3CA *were also detected in epidermal nevi (EN) and seborrheic keratoses (SK), two benign skin lesions [14]. All mutations detected in EN samples were of the E545G type whereas SK displayed E542K, E545K and H1047R mutations.

PCR-based screening methods (e.g. single strand conformation polymorphism analysis, high resolution melting analysis, ARMS/Scorpion assays) and direct sequencing of PCR products have typically been applied to the identification of *PIK3CA *mutations [5,13,15,16]. However, with large numbers of samples, these approaches are time consuming and/or expensive. As specific inhibitors for PIK3CA become available it will be essential to be able to screen patient samples rapidly. Primer extension (SNaPshot) assays have been developed for several genes with common mutations, e.g. *FGFR3 *and *BRCA1/2 *[17,18]. The SNaPshot method offers a specific, sensitive, inexpensive and rapid alternative to screening for mutations and subsequent confirmation by sequencing. Here we describe a SNaPshot assay for the simultaneous detection of the *PIK3CA *mutations, E542K, E545G, E545K and H1047R.

## Methods

### Samples and DNA extraction

Sixteen bladder tumour-derived cell lines (5637, 253J, 639V, 647V, 97-21, 97-24, 97-29, BFTC909, CAL29, HT1197, HT1376, J82, JO'N, TCC-SUP, VMCUB1, VMCUB3), 175 fresh-frozen bladder tumour tissue samples and 5 formalin-fixed paraffin-embedded seborrheic keratosis samples were used. All DNAs were extracted using a QIAmp DNA kit.

### Multiplex PCR primers and SNaPshot probes

Multiplex PCR primers and SNaPshot probes were selected using a web-based oligonucleotide check tool  so that they had matching melting temperatures of approximately 65°C. Primer sequences were analysed for secondary structures, complementarity and specificity. Primers were selected for amplification of exon 9 (ex9-Fw 5'-AGTAACAGACTAGCTAGAGA-3'; ex9-Rv 5'-ATTTTAGCACTTACCTGTGAC-3') and exon 20 (ex20-Fw 5'-GACCCTAGCCTTAGATAAAAC-3'; ex20-Rv 5'-GTGGAAGATCCAATCCATTT-3'), with the amplicons covering hotspot codons 542, 545 and 1047. SNaPshot probes for detection of E542K, E545G, E545K and H1047R mutations were designed to anneal on the sense strand immediately adjacent to the mutation site (Table 1). Each probe was synthesised with a different length of poly(dT) tail to allow separation of SNaPshot products on the basis of size (Table 1).

**Table 1:** SNaPshot probes for the detection of *PIK3CA *mutations.

**Probe**	**Sequence (5'->3')**	**Size (bp)**	**Mutation**	**Concentration** **in probe mix (μM)**
E542K	T_(19)_TACACGAGATCCTCTCTCT	38	G->A	0.8
E545G	T_(29)_TCCTCTCTCTGAAATCACTG	49	A->G	2.3
E545K	T_(34)_ATCCTCTCTCTGAAATCACT	54	G->A	1.5
H1047R	T_(46)_TGAAACAAATGAATGATGCAC	67	A->G	1.5

### Multiplex PCR amplification

Multiplex PCR was performed in a volume of 15 μl containing 1 × PCR buffer, 1.5 mM MgCl_2_, 0.17 mM dNTPs, 0.7 μM of each primer, 5% glycerol, 1 unit GoTaq DNA polymerase and 20 ng of template DNA. Thermal cycler conditions were: 95°C for 5 min, 35 cycles of 95°C for 45 sec, 60°C for 45 sec, 72°C for 45 sec and finally 10 min at 72°C. The number of cycles was increased to 45 for the analysis of DNAs extracted from paraffin-embedded material. Multiplex PCR products were checked for quality and yield by running 3 μl in 2% agarose-TBE gels. The remaining PCR products were treated with 3 units of shrimp alkaline phosphatase and 2 units of exonuclease I to remove excess deoxyribonucleotide triphosphates (dNTPs) and primers, respectively.

### SNaPshot analysis

SNaPshot analysis was performed using an Applied Biosystems SNaPshot Multiplex Kit. Reactions were performed in a volume of 9 μl containing 2.5 μl of SNaPshot Ready Multiplex Ready Reaction Mix, 1 × BigDye sequencing buffer, 1 μl of probe mix (see Table 1 for probe concentrations) and 1 μl of shrimp alkaline phosphatase/exonuclease-treated multiplex PCR product. Extension reactions were performed in a thermal cycler and consisted of 35 cycles of denaturation at 95°C for 10 sec and annealing/extension at 58.5°C for 40 sec. Labelled extension products were treated with shrimp alkaline phosphatase (1 unit per sample) then diluted 1 in 10. 1 μl of the diluted extension product was mixed with 9.8 μl of HiDi™ formamide and 0.2 μl of Genescan-120LIZ size standard. Products were denatured at 95°C for 5 minutes then separated using an ABI PRISM 3100 Genetic Analyzer with a 36 cm length capillary and POP-7™ polymer. Analysis was performed using GeneMapper 3.7 Software.

### Sensitivity of the SNaPshot assay

Mixtures of bladder tumour DNAs heterozygous for *PIK3CA *mutations and normal DNA were prepared to test the sensitivity of the SNaPshot assay. 20 ng of template DNA containing mutant DNA at final percentages of 100%, 25%, 10%, 5% or 1% was used in SNaPshot analysis.

## Results and discussion

The first step of the SNaPshot assay described here involved the design and optimisation of a multiplex PCR reaction for the simultaneous amplification of DNA fragments from exons 9 and 20. Relatively small PCR amplification product sizes (139 bp for exon 9 and 109 bp for exon 20) were chosen so that the assay could be applied to the analysis of partially degraded DNA samples such as those obtained from formalin-fixed paraffin-embedded samples. PCR primers were initially tested in single-product reactions prior to being used in multiplex reactions. Similar quantities of both products were observed in multiplex PCR reactions.

The primer extension (SNaPshot) assay utilises an Applied Biosystems SNaPshot Multiplex Kit that contains a reaction mix of four differentially fluorescently labelled ddNTPs, allowing the interrogation of each base at a mutation site. When used in combination with the size-specific *PIK3CA *SNaPshot probes (Table 1), it was possible to screen simultaneously for E542K, E545G, E545K and H1047R mutations. Initially, extension reactions were performed using equal concentrations of the probes. Subsequently, the concentration of each probe was adjusted to give more comparable peak heights (Table 1).

**Figure 1:**
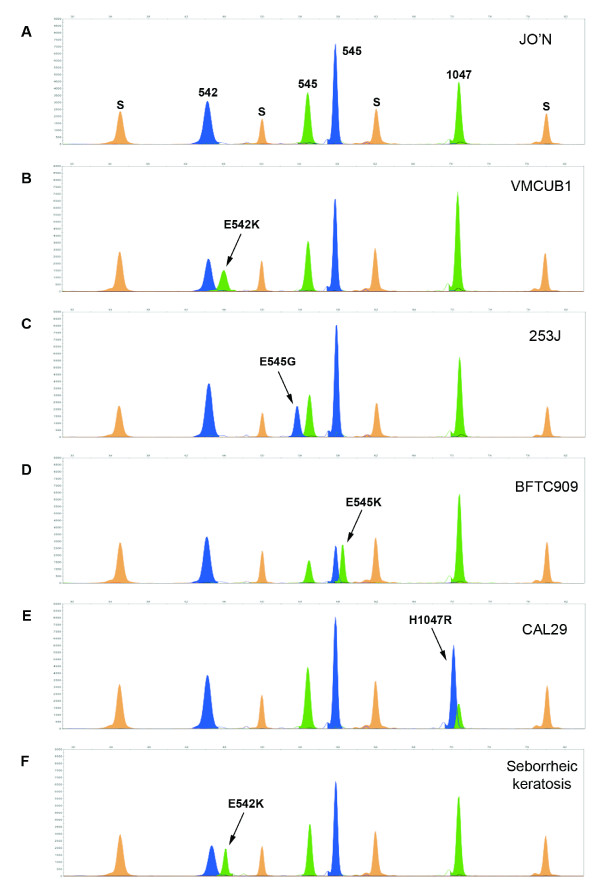
**SNaPshot detection of four common hotspot mutations in the *PIK3CA *gene**. (**A-E**) SNaPshot results obtained from the analysis of bladder tumor-derived cell line DNAs illustrating (**A**) wildtype, (**B**) E542K, (**C**) E545G, (**D**) E545K and (**E**) H1047R electropherogram patterns. (**F**) SNaPshot detection of an E542K codon mutation in DNA extracted from a formalin-fixed paraffin embedded seborrheic keratosis sample. Bases are represented by the following colours: A = green; C = black; G = blue; T = red. Orange peaks (S) represent the internal Genescan-120LIZ size standards. All DNAs were extracted using a QIAmp DNA kit.

To evaluate the performance of the SNaPshot assay we performed a blind screen of 16 bladder tumor-derived cell line and 175 fresh-frozen primary bladder tumor DNAs that had previously been analysed for *PIK3CA *mutations in exons 9 and 20 by high-resolution melting analysis using the LightScanner system and direct sequencing (Platt *et al*. manuscript in preparation). SNaPshot detected heterozygous mutations in 7 cell lines (E542K n = 1 [VMCUBI]; E545G n = 1 [253J]; E545K n = 4 [HT1197; BFTC909; VMCUB3; TCC-SUP]; H1047R n = 1 [CAL29]) and 37 tumors, and results were 100% concordant with the data obtained by Platt *et al*. A representative example of each of the four common codon mutations detected in the cell lines is shown in Figure 1(A–E). Peaks are colour-coded by the Genemapper software according to the dye-label on the incorporated ddNTP. Genotype scoring is manual and is based on the peak colour and position relative to a set of internal size standards. Mutations in each codon were easily identified on the basis of peak size and colour. Slight shifts in the molecular weights of mutant alleles were observed due to mobility differences in the fluorophores used to detect each of the bases. However, probes were sufficiently spaced such that these mobility differences did not impair interpretation of the resulting electropherograms.

We also applied the method to a panel of five DNAs extracted from formalin-fixed paraffin-embedded seborrheic keratoses. When analysing samples of this nature it was observed that the quality of SNaPshot results obtained was invariably linked to the intensity of the multiplex PCR products. To obtain sufficient multiplex PCR product for SNaPshot analysis, the number of PCR amplification cycles was therefore increased to 45 for these samples. Screening revealed an E542K mutation in one of the DNAs (Figure 1F) whilst all other samples were found to be wildtype, confirming results obtained previously by direct sequencing [19].

**Figure 2:**
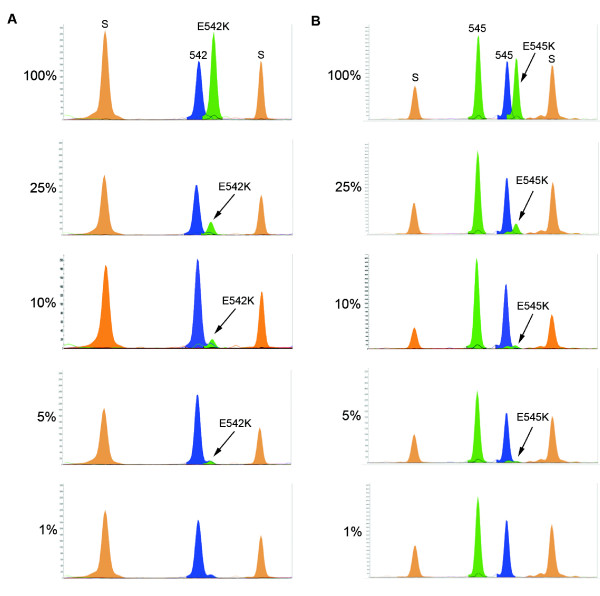
**Sensitivity of the *PIK3CA *SNaPshot assay**. SNaPshot could detect mutant *PIK3CA *when it represented 5% to 10% of the total DNA present. Representative examples for bladder tumors with (**A**) E542K and (**B**) E545K are shown.

Finally, we assessed the sensitivity of the SNaPshot assay by mixing heterozygous mutant DNA from low grade/stage bladder tumors (that are commonly diploid or near-diploid) with normal DNA in varying proportions. We were able to detect mutations E542K, E545K and H1047R when mutant DNA represented 5–10% of the total input DNA. Figure 2 shows representative examples of results obtained for codons E542K and E545K. As mutant DNAs used in these experiments were heterozygous, the observed sensitivity results equate to being able to detect one mutant allele in a background of 39 (5%) or 19 (10%) wildtype alleles. This level of detection is comparable to that reported for the FGFR3 SNaPshot assay of van Oers *et al*. [17]. We were also able to achieve a 5% level of detection for codon E545K when mutant DNAs from two triploid bladder tumor cell lines (TCC-SUP and BFTC909) were used (data not shown). Calling the presence of low-level mutations is more difficult and in these cases more than one individual should perform scoring and the assay should be repeated to confirm that the mutations do not represent artefacts.

It is perhaps surprising that we did not uncover extra mutations to those detected by the LightScanner and direct sequencing approach. Board *et al*. [15] reported that conventional sequencing was unable to detect the presence of H1047R and E542K mutations when present at <50% and <30% of the total mixture, respectively. On this basis, the SNaPshot assay should be more sensitive than the LightScanner and direct sequencing confirmation approach. The high concordance of results most likely reflects the fact that the DNAs analysed in our study were extracted from samples consisting of >80% tumor cells.

The SNaPshot assay described in the present study represents a relatively inexpensive approach to the detection of *PIK3CA *mutations. The cost of the SNaPshot Multiplex Ready Reaction Mix is £1.16 per sample. In comparison a standard sequencing reaction using BigDye Terminator v1.1 Ready Reaction Mix costs £2.59 per sample. Methods that employ pre-screening approaches may initially represent a cheaper alternative to mutation detection but the subsequent requirement for sequence verification of potential mutations significantly increases the time and cost of these assays relative to the SNaPshot method. The *PIK3CA *SNaPshot assay is less expensive than methods which employ the use of fluorescently-labelled oligonucleotide probes [15] as the probes used in the SNaPshot assay are unlabelled primers. Pati *et al*. [20] compared the SNaPshot method to pyrosequencing and biplex invader SNP genotyping methods. The authors concluded that the SNaPshot assay required little optimisation and was comparable to these two methods but with a higher cost when singleplex reactions were used. The authors also reported that failed reactions were mostly associated with poor PCR amplification. In the present study, we have designed a robust multiplex PCR and did not observe any such reaction failures. The combined multiplex PCR and SNaPshot reaction makes it possible to screen simultaneously for four mutations. In addition, all steps of the SNaPshot assay are performed in 96 well plates and the method could easily be automated for high throughput analysis.

## Conclusion

A SNaPshot assay for the simultaneous screening of four frequent *PIK3CA *codon mutations has been developed. The detection of multiple mutations in a single reaction reduces cost, amount of patient sample DNA and sample handling required. The interpretation of results is quick and easy and the method is flexible so that if required, appropriately sized probes targeting additional hotspot codons could be added to the assay. A major advantage is the avoidance of an extra sequencing step that is a requirement of many pre-screening-based methods.

As specific inhibitors for PIK3CA become available, rapid screening of patient samples for mutations will be essential. The SNaPshot assay described here can be high throughput and is robust and objective making it suitable for use in such a diagnostic setting.

## Competing interests

The authors declare that they have no competing interests.

## Authors' contributions

CDH, TZ, EZ, CH and MK participated in the conception and design of the study, and writing of the manuscript. CDH and CH performed SNaPshot experiments and data analysis. All authors read and approved the final manuscript.
